# Delimiting the Function of the C-Terminal Extension of the *Escherichia coli* [NiFe]-Hydrogenase 2 Large Subunit Precursor

**DOI:** 10.3389/fmicb.2019.02223

**Published:** 2019-09-24

**Authors:** Constanze Pinske, Claudia Thomas, Kerstin Nutschan, R. Gary Sawers

**Affiliations:** Institute of Microbiology, Martin-Luther University Halle-Wittenberg, Halle (Saale), Germany

**Keywords:** hydrogenase, Hyp proteins, large-subunit precursor, protein interaction, protease, maturation

## Abstract

The active site of all [NiFe]-hydrogenases (Hyd) has a bimetallic NiFe(CN)_2_CO cofactor that requires the combined action of several maturation proteins for its biosynthesis and insertion into the precursor form of the large subunit of the enzyme. Cofactor insertion is an intricately controlled process, and the large subunit of almost all Hyd enzymes has a C-terminal oligopeptide extension that is endoproteolytically removed as the final maturation step. This extension might serve either as one of the recognition motifs for the endoprotease, as well as an interaction platform for the maturation proteins, or it could have a structural role to ensure the active site cavity remains open until the cofactor is inserted. To distinguish between these alternatives, we exchanged the complete C-terminal extension of the precursor of *Escherichia coli* hydrogenase 2 (Hyd-2) for the C-terminal extension of the Hyd-1 enzyme. Using in-gel activity staining, we demonstrate clearly that this large subunit precursor retains its specificity for the HybG maturation chaperone, as well as for the pro-HybC-specific endoprotease HybD, despite the C-terminal exchange. Bacterial two-hybrid studies confirmed interaction between HybD and the pro-HybC variant carrying the exchanged C-terminus. Limited proteolysis studies of purified precursor and mature HybC protein revealed that, in contrast to the precursor, the mature protein was protected against trypsin attack, signifying a major conformational change in the protein. Together, our results support a model whereby the function of the C-terminal extension during subunit maturation is structural.

## Introduction

A still unresolved issue on the maturation pathway of the catalytic, or large, subunit of [NiFe]-hydrogenases (Hyd) is what role the C-terminal oligopeptide extension plays during the maturation process: Does it act as a recognition motif allowing interaction with maturation proteins, or does it function as a form of “intra-molecular” chaperone that constrains the large subunit precursor in a conformation allowing access of the maturation machinery, which then interacts elsewhere on the protein? Or does it do both?

**Figure 1:**
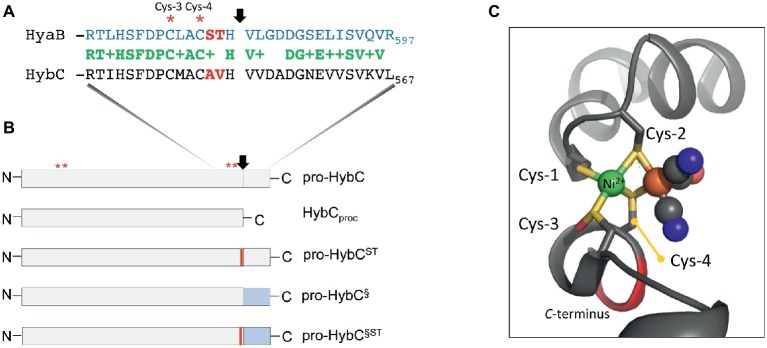
Schematic representation of the HybC large subunit variants. **(A)** The amino acid sequences in single-letter code of the C-terminal 30 residues of the large subunit pro-HybC and pro-HyaB precursors are shown. Identical amino acids are shown in green, the + indicates similar amino acids and the residues depicted in red signify those exchanged in this study. The vertical arrow indicates the site of endoproteolytic cleavage and the red asterisks signify the conserved Cys-3 and Cys-4 residues involved in coordinating the Ni-Fe-cofactor. **(B)** The HybC variants used in this study (not drawn to scale) are shown, as is the location of the sequence represented in part **(A)**. N and C represent N- and C-terminus of the polypeptides, respectively. The thin red bar indicates that A and V were changed to S and T, and the blue rectangle signifies that the 15-amino acid extension (after the cleavage site) from pro-HyaB replaced the native oligopeptide. The red asterisks depict the approximate locations of the four conserved Cys residues (1–4) that coordinate the NiFe-cofactor. **(C)** The structural organization of the Cys-1 through Cys-4 and how they coordinate the NiFe(CN)_2_CO group in HybC (Beaton et al., 2018) is shown for the reduced enzyme (6EHS). The representation was generated using PyMOL. The green sphere represents the nickel and the large brown sphere the iron ion, where the cyanyl (black and blue spheres) and carbonyl (black and red spheres) ligands are coordinated.

With the exception of the large subunit of sensory and Ech hydrogenases (Theodoratou et al., 2005), the final step in the maturation process of the large subunit involves endoproteolytic cleavage of an approximately 15-amino acid-long oligopeptide from the C-terminus of the protein (Gollin et al., 1992; Menon et al., 1993; Rossmann et al., 1994; Böck et al., 2006). Proteolytic cleavage of the precursor of the large subunit is an exquisitely controlled process and only occurs after insertion of the bimetallic NiFe-cofactor has been completed (Böck et al., 2006; Sawers and Pinske, 2017). The NiFe-cofactor is coordinated within the active site by four conserved cysteinyl thiolates (henceforth referred to as Cys1–4), two of which are located N-terminally (Cys1–2) and the other two (Cys3–4) are close to the ultimate C-terminus of the protein (see Figure 1; Magalon and Böck, 2000).

Having been constructed on the HypCDEF protein scaffold complex (Böck et al., 2006; Sawers and Pinske, 2017), the Fe(CN)_2_CO moiety of the cofactor is the first component to be delivered to the precursor, probably by the HypC protein. In bacteria synthesizing more than one Hyd, paralogues of HypC exist, e.g., HybG in *Escherichia coli*, and these paralogues deliver the Fe(CN)_2_CO moiety to a specific Hyd enzyme (Blokesch et al., 2001). Only after the Fe group has been delivered and coordinated by the Cys residues 1 and 2, can nickel be delivered by the combined actions of the HypAB and SlyD proteins (Lacasse and Zamble, 2016). The nickel is initially coordinated by Cys-3 (Figure 1A). Nickel is the template recognized by the specific endoprotease (Theodoratou et al., 2000a) and cleavage occurs between the conserved His and Val residues, which are located three and four amino acid residues, respectively, C-terminal to the Cys-4 residue that coordinates the cofactor (Figure 1A). Based on the concept of Schechter and Berger (1967) and Hedstrom (2002), these two positions of a peptide (P1 and P1′) determine the specificity of the protease. Notably, however, the Hyb endoprotease cannot be classified either as a metallo- or as a serine-protease (Rossmann et al., 1995; Sawers, 2012). Proteolytic removal of the C-terminal oligopeptide causes a conformational switch in the complete protein bringing the Cys-4 residue (Figures 1A,C) into coordination distance to bridge both metals and “fix” the cofactor firmly within the active site (Magalon and Böck, 2000; Böck et al., 2006; Kwon et al., 2018).

The recently determined crystal structure of the HyhL large subunit precursor of the Hyd enzyme from the hyperthermophilic archaeon *Thermococcus kodakarensis* in complex with the nickel delivery protein HypA provided an initial indication that the presence of the C-terminal extension causes significant displacement of Cys-4 from the other three coordinating Cys residues, leaving the active site accessible for cofactor delivery (Kwon et al., 2018). Moreover, HypA was shown to bind at a flexible region of the large subunit near the N-terminus, suggesting that the C-terminus is not involved in binding maturation proteins.

Our model system to study the role of the C-terminal extension in maturation is the large subunit, HybC, of *E. coli* Hyd-2. Hyd-2 enzyme activity is readily detectable and an array of available mutants makes this system highly amenable to analysis of the maturation process (Pinske et al., 2015; Thomas et al., 2015). Moreover, the recently determined crystal structure of the enzyme reveals that the mature HybC subunit has a compact and characteristic oxidoreductase fold common to many hydrogenases (Beaton et al., 2018).

HybG, a HypC paralogue, is specifically required for maturation of pro-HybC (Blokesch et al., 2001), the precursor, or proprotein, form of HybC and interacts with this polypeptide, but not with the processed form of the protein HybC_proc_ (Thomas et al., 2015; Senger et al., 2017). HybC_proc_ is a genetically generated variant of pro-HybC (Figure 1B) that lacks the 15-amino acid C-terminal extension normally removed by the HybC-specific endoprotease HybD *in vivo* (Fritsche et al., 1999; Theodoratou et al., 2005; Sawers, 2012). Preliminary studies carried out by our group have indicated that when the terminal 15 amino acids present on pro-HybC are exchanged for those of the *E. coli* Hyd-1 large subunit precursor, pro-HyaB (see Figure 1B), cleavage of the hybrid proprotein retains its specificity for the HybD endoprotease and does not become dependent on HyaD, the pro-HyaB-specific endoprotease (Thomas et al., 2015). An alignment of the C-terminal regions of pro-HybC and pro-HyaB reveals that the 15-amino acid extension of both proteins shares 7 conserved and 5 similar amino acid residues (Figure 1A). Overlooked in the initial study by Thomas et al. (2015) were the two amino acid residues immediately adjacent to Cys-4, which are AV in HybC and ST in HyaB (Figure 1A). These residues locate to the P2 and P3 positions that still have a significant impact on protease recognition (Schechter and Berger, 1967; Hedstrom, 2002). Because of the important role played by Cys-4 in coordinating the NiFe-cofactor (Magalon and Böck, 2000) and the significant displacement of this residue revealed by the crystal structure of immature HyhL (Kwon et al., 2018), this suggests a potentially significant role for residues A-550 and V-551 in the recognition of the precursor by hydrogenase maturation proteins. We therefore decided to analyze whether these two amino acid residues act as determinants in the recognition of pro-HybC by either HybG or HybD. Our findings indicate that, in contrast to the latter suggestion, the complete C-terminal extension behind the final cysteinyl residue coordinating the cofactor functions solely to constrain pro-HybC in a conformation appropriate for interaction with the maturation machinery.

## Materials and Methods

### Strains and Growth Conditions

The strains listed in Table 1 were used in this study. All growth experiments involving the analysis of in-gel hydrogenase enzyme activity were performed anaerobically in M9 minimal medium (Sambrook et al., 1989) supplemented with 0.1 mM CaCl_2_, 0.1 mM thiamin dichloride, and trace element solution SLA (Hormann and Andreesen, 1989) at 37°C in Hungate tubes under a nitrogen gas atmosphere as described (Thomas et al., 2015). The carbon source was glucose (0.8% w/v). For routine cloning experiments, strains were grown on LB-agar plates or in LB-broth at 37°C (Miller, 1972). When required, the antibiotics ampicillin, kanamycin, or chloramphenicol were added to a final concentration of 100, 50, or 25 μg ml^−1^, respectively. Cells were harvested by centrifugation at 5,000 *g* for 15 min at 4°C when cultures had reached an OD_600 nm_ of between 0.8 and 1.2. Cell pellets were either used immediately or stored at −20°C until use.

**Table 1:** Strains and plasmids used in this study.

Strain or plasmid	Relevant genotype or characteristic(s)	Reference or source
**Strain**		
BTH101	F^−^, *cya*-99, *araD*139, *galE*15, *galK*16, *rpsL*1(Str^R^), *hsdR*2, *mcrA*1, *mcrB*1	Karimova et al., 1998
MC4100	F^−^ *araD139* (*argF-lac*)*U169 ptsF25 deoC1 relA1 flbB5301 rspL150*	Casadaban, 1976
FTD150	As MC4100, but Δ*hyaB* Δ*hybC* Δ*hycE* Δ*hyfB-R*	Redwood et al., 2008
CB16	As FTD150, but *hyaB^+^* Δ*hyaD*::Kan^R^	Thomas et al., 2015
CB17	As FTD150, but *hybC^+^* Δ*hybD*::Kan^R^	This study
CB18	As FTD150, but *hybC^+^* Δ*hybG*::Kan^R^	This study
CB19	As FTD150, but *hycE^+^* Δ*hypC*::Kan^R^	This study
**Plasmids**		
pASK-hybC	*hybC* in pASK-IBA5^+^, Amp^R^	Pinske et al., 2011
pASK-hybC_proc_	pASK-hybC, V553 Stop (codon 553 converted to TAA), Amp^R^	Thomas et al., 2015
pASK-hybC^hyaB^	*hybC* with *hyaB C*-Terminus in pASK-IBA5^+^, Amp^R^; product designated pro-HybC^§^	Thomas et al., 2015
pASK-hybC^ST^	*hybC* with AV changed to ST in pASK-IBA5^+^, Amp^R^; product designated pro-HybC^ST^	This study
pASK-hybC^hyaBST^	*hybC* with *hyaB C*-Terminus and AV changed to ST in pASK-IBA5^+^, Amp^R^; product designated pro-HybC^§ST^	This study
pASK-hybG	*hybG* in pASK-IBA3, carries a C-terminal Strep-tag; Amp^R^	Soboh et al., 2013
pCB-hybC	pCAN-hybC (Kitagawa et al., 2005) with a TAA at codon 568 in *hybC*, producing His-pro-HybC; Cm^R^	This study
pCB-hybC_proc_	pCAN-hybC with a TAA at codon 553 (GTA → TAA) in *hybC*, producing His-HybC_proc_; Cm^R^	This study
pUT18	Bacterial two hybrid plasmid expressing the T18 fragment and a MCS at the 5′ end of T18, Amp^R^	Karimova et al., 1998
pKT25	Bacterial two hybrid plasmid expressing the T25 fragment and a MCS at the 3′ end of T25, Cm^R^	Karimova et al., 1998
pT18-zip	pUT18, Leucine zipper fused to T18 fragment (225-399 amino acids of CyaA)	Karimova et al., 1998
pT25-zip	pKT25, Leucine zipper fused to T25 fragment (1-224 amino acids of CyaA)	Karimova et al., 1998
pT18-hybD	pUT18, *hybD*^+^, Amp^R^	This study
pT25-hybC	pKT25, *hybC*^+^, Cm^R^	This study
pT25-hybC^§^	pKT25, *hybC* with *hyaB C*-terminus, Cm^R^	This study
pT25-hybC^ST^	pKT25, *hybC*^+^ including the AV550ST exchange, Cm^R^	This study
pT25-hybC^§ST^	pKT25, *hybC* with *hyaB C*-terminus and including the AV550ST exchange, Cm^R^	This study
pT25-hyaB	pKT25, *hyaB*^+^, Cm^R^	This study
pT25-hyaB^§^	pKT25, *hyaB* with *hybC C*-terminus, Cm^R^	This study
pT-hypC	pT7–7 *hypC*^+^, HypC carries a Cterminal Strep-tag, Amp^R^	Soboh et al., 2013

### Plasmid and Strain Construction

Amino acid exchanges of the A550S and V551 T (numbers are based on untagged protein) were introduced simultaneously in *hybC* using the NEBase changer method (NEB, USA). The templates used were the plasmids pASK-hybC (Pinske et al., 2011) and pASK-hybC^hyaB^ (Thomas et al., 2015). The oligonucleotides used were wtHybC_AV>ST_FW with HybC_AV>ST_RW for the pASK-hybC plasmid and HybC/HyaB_AV>ST_FW with HybC_AV>ST_RW for the pASK-hybC^hyaB^ plasmid (Table 2). The stop codons introduced into *hybC* in pCAN-hybC (Table 1; Kitagawa et al., 2005) to generate pCB-hybC and pCB-hybC_proc_ (see Table 1) were done using PCR mutagenesis with pCAN-hybC as DNA template and the oligonucleotides described previously (Thomas et al., 2015).

**Table 2:** Oligonucleotides used in this study.

Oligonucleotide	Sequence 5′ → 3′
wtHybC_AV>ST_FW	CATGGCCTGTTCAACACACGTAGTGGATG
HybC/HyaB_AV>ST_FW	CATGGCCTGTTCAACACACGTGCTGG
HybC_AV>ST_RW	CACGGGTCAAAGGAGTGA
HyaB_FW_PstI	GCGCTGCAGGGAGCACTCAGTACGAAACTC
HyaB_RW_BamHI	GCGGGATCCTTAACGCACCTGCACGGAGATC
HybC_FW_PstI	GCGCTGCAGGGAGCCAGAGAATTACTATTG
HybC_RW_BamHI	GCGGGATCCTTACAGAACCTTCACTGAAAC
HybD_FW_HindIII	GCGAAGCTTATGCGTATTTTAGTCTTAGG
HybD_RW_EcoRI	GCGGAATTCGAGTCATGAATCGCCTCCCGTG

Plasmids for the bacterial-two-hybrid analysis were constructed by using either the large subunit variants as template or, for the *hybD* gene, chromosomal DNA as template. The genes were amplified with Q5 polymerase (NEB, USA) using the appropriate combination of oligonucleotides (Table 2), and PCR fragments were subsequently digested with PstI and BamHI for the large subunits or with HindIII and EcoRI for *hybD* and ligated into the bacterial two hybrid vectors pKT25 or pUT18 (Karimova et al., 1998), which had been previously digested with the same respective enzyme combinations. The authenticity of all constructs was verified by DNA sequencing.

*E. coli* strains were constructed using P1*kc*-mediated phage transduction (Miller, 1972) to introduce the respective defined deletion mutation in *hypC* or *hybG* from the appropriate donor strain of the Keio collection (Baba et al., 2006).

### Preparation of Crude Cell Extracts, Non-denaturing Polyacrylamide Gel Electrophoresis, and Hydrogenase Activity-Staining

Crude cell extracts were prepared anaerobically as described (Pinske et al., 2012). Determination of protein concentration was done as described (Lowry et al., 1951). Non-denaturing polyacrylamide gel electrophoresis (PAGE) was performed anaerobically. Separating gels included 0.1% (w/v) Triton X-100 as described (Ballantine and Boxer, 1985). Crude extracts were incubated with a final concentration of 4% (w/v) Triton X-100 prior to application (usually 50 μg of protein) to the gel, which included 6% (w/v) polyacrylamide. Hydrogenase activity-staining was done in 50 mM MOPS buffer pH 7.0, as described (Pinske et al., 2012) and included 0.5 mM BV and 1 mM 2,3,5-triphenyltetrazolium chloride (TTC). Gels were incubated under an atmosphere of 100% hydrogen gas.

### Purification and Limited Proteolysis of Pro-HybC and HybC_proc_

N-terminally His-tagged HybC variants were purified after overproduction in strain CB17 (Table 1) by metal-affinity chromatography using cobalt-charged TALON^®^ superflow resin (Clontech Laboratories Inc., USA). A column containing 1 ml bed-volume of resin was pre-equilibrated with 10 ml of anaerobic buffer A (50 mM Tris–HCl, pH 8, 300 mM NaCl) followed by application of filtered (0.45 μm sterile filter, Sartorius), anaerobically prepared crude extract (typically 5 ml, 10 mg ml^−1^ protein) containing the His-tagged protein at a flow rate of 1 ml min^−1^. Unbound proteins were washed from the column with 10–15 column volumes of buffer A, followed by a washing step (3 column volumes) using buffer A containing 30 mM imidazole. Bound, His-tagged proteins were eluted with buffer A containing 300 mM imidazole. After elution, pooled eluate containing the purified protein(s) of interest was buffer-exchanged into buffer C (25 mM Tris-HCl, pH 8, 150 mM NaCl) using a Sephadex PD-10 column (GE Healthcare, Germany).

Aliquots (100 μg) of pro-HybC or HybC_proc_ (both N-terminally His-tagged) in a 100 μl reaction volume of 200 mM HEPES pH 7.8 containing 200 mM NaCl and 40 mM CaCl_2_ were incubated with 1 μg of trypsin or chymotrypsin and 10 μl aliquots were removed at the indicated times and immediately added to boiling SDS buffer to terminate the reaction. Polypeptides were separated in denaturing SDS-PAGE (Laemmli, 1970) containing 12.5% (w/v) polyacrylamide. Gels were subsequently stained with Coomassie Brilliant Blue.

C-terminally Strep-tagged HypC and HybG were purified as described in Soboh et al. (2011, 2013). Aliquots of 50 μg were used in limited proteolysis experiments.

### Analysis of Protein Interactions Using the Bacterial Two-Hybrid System

These experiments were performed as described by Pinske (2018). Briefly, growth of strain BTH101 (Karimova et al., 1998) transformed with two compatible BTH plasmids was performed under anaerobic conditions in TGYEP medium (1% w/v tryptone, 0.5% w/v yeast extract, 0.8% w/v glucose, 0.1 M potassium phosphate buffer pH 6.5; Begg et al., 1977) at 30°C for 16 h in the presence of ampicillin and chloramphenicol. Determination and calculation of β-galactosidase enzyme activity was done using a 0.5 ml culture grown according to Miller (1972). Each experiment was performed in biological, as well as technical, triplicates. In a qualitative test, 5 μl aliquots of culture were dropped on MacConkey plates containing 0.5% (w/v) maltose, ampicillin, and chloramphenicol. The plates were incubated aerobically at 30°C for 16 h and strains lacking β-galactosidase enzyme activity remained white, while those exhibiting activity ranged from pink to deep red (data not shown).

## Results and Discussion

### Exchange of the Amino Acids Ala-550 and Val-551 on Pro-HybC for Those in Pro-HyaB Does Not Prevent Processing

**Figure 2:**
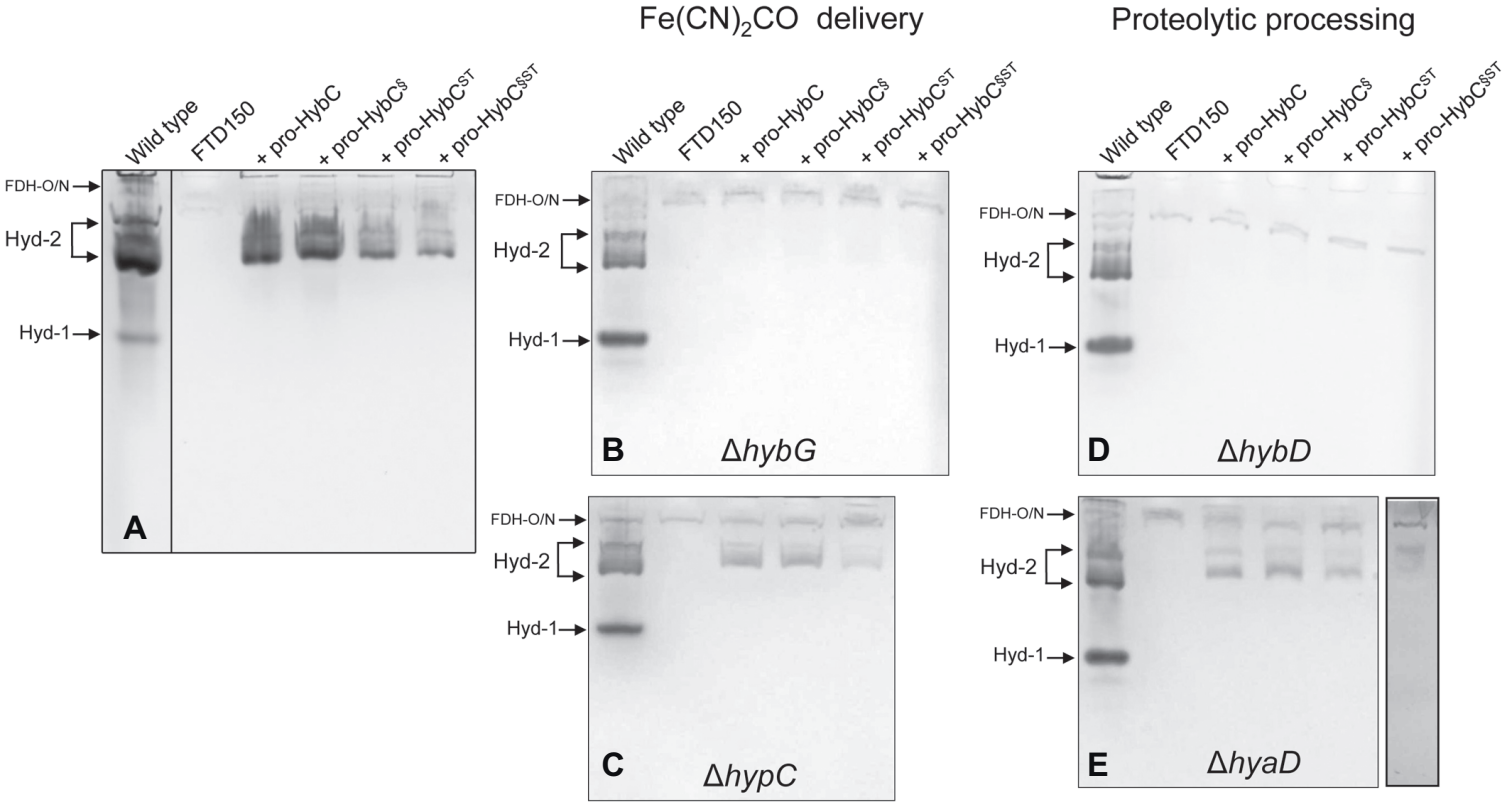
Hyd-2 enzyme activity profiles of the different HybC variants. Activity-stained native polyacrylamide gels are shown after anaerobic growth of the indicated strains in minimal-glucose medium (see Section “Materials and Methods” for details). The locations of the respective H_2_-oxidizing enzyme complexes are indicated on the left of each panel. Each panel has a positive control (wild type = MC4100) and a negative control (FTD150 Δ*hyaB*, *hybC*, *hycE*, and Δ*hyfB-R*). The four lanes on the right of each panel represent the gene products encoded by the corresponding plasmids introduced into FTD150 (panel **A**), CB18 (Δ*hybG*; panel **B**), CB19 (Δ*hypC*; panel **C**), CB17 (Δ*hybD*; panel **D**), or CB16 (Δ*hyaD*; panel **E**). All other genes necessary for synthesis of an active Hyd-2 enzyme are present in each strain, except in strains CB17 and CB18. Note that in panel **(A)**, the extract derived from MC4100 was run on a separate gel, as was the final lane in panel **(E)**. These experiments were repeated on minimally three separate occasions.

The C-terminal peptide on pro-HybC, the large subunit precursor of Hyd-2, functions either as a recognition motif for the Hyp cofactor-insertion machinery or it maintains the empty active site in an open conformation, facilitating insertion of the nickel and Fe(CN)_2_CO moieties of the cofactor. Current evidence strongly suggests the latter role (Thomas et al., 2015; Kwon et al., 2018). To demonstrate this conclusively, however, we decided to generate a HybC variant carrying a complete swap of all 18 amino acids after Cys-4 (Figure 1A), for the corresponding C-terminal 18-amino acid oligopeptide from the Hyd-1 large subunit precursor pro-HyaB (Figure 1B). A variant in which the final 15-amino acid residues of the pro-HybC protein were exchanged for those of pro-HyaB was constructed previously (Thomas et al., 2015). This construct, referred to in this study as pro-HybC^§^ (Figure 1B), was used as the basis to introduce further changes at codons 550 and 551 in the *hybC* gene, resulting in a protein product (pro-HybC^§ST^) carrying the Hyd-1-specific ST instead of the Hyd-2-specific AV amino acid residues at these positions (Figure 1A). These exchanges were also made in the native *hybC* gene, delivering pro-HybC^ST^ (Figure 1B). The plasmids delivering the gene products pro-HybC, pro-HybC^§^, pro-HybC^ST^, and pro-HybC^§ST^ (see Table 1) were introduced into the *E. coli* strain FTD150 (Redwood et al., 2008), which lacks the genes encoding the large subunits of all four hydrogenases, as well as into isogenic strains lacking *hybG* (CB18), *hypC* (CB19), *hyaD* (CB16), or *hybD* (CB17). After anaerobic growth in minimal medium with glucose as a carbon source, cell-free extracts were prepared, and the Hyd-2 enzyme activity profile of each strain was analyzed after separation of enzyme complexes in native-PAGE (Figure 2). The wild-type strain MC4100 acted as a positive control in each experiment and revealed three sets of signals (Ballantine and Boxer, 1985; Pinske et al., 2011) representing H_2_-oxidizing enzyme activity (see Figure 2A), characteristic for the conditions used: Hyd-1 had the fastest mobility in the gel; Hyd-2 migrated as a double band, and occasionally a third band could be discerned; and near the origin of the gel a weakly staining activity band due to a secondary H_2_-oxidizing activity of the formate dehydrogenases FDH-O and FDH-N was observed (Soboh et al., 2011). The activity due to FDH-O/N acted as a useful loading control in these experiments and was the only activity band visible in extracts of the negative control FTD150 lacking a plasmid (Figure 2). Introduction of the genes encoding pro-HybC and pro-HybC^§^ restored Hyd-2 activity to strain FTD150, indicating that both precursor proteins were efficiently recognized and processed to active subunits by the Hyp maturation machinery (Thomas et al., 2015). Exchange of amino acids AV for ST at amino acid positions 550-551, delivering pro-HybC^§ST^, also resulted in an active Hyd-2 enzyme, albeit with a slightly weaker activity signal (Figure 2A). This result indicated that exchange of the AV for ST amino acid residues adjacent to the terminal Cys-549 residue only reduced the efficiency of maturation or processing of the large subunit precursor but did not prevent maturation of an active Hyd-2 enzyme. Exchange of the complete 18-amino acid C-terminal peptide on pro-HybC for that on pro-HyaB, delivering pro-HybC^§ST^, reduced the level of Hyd-2 activity still further under the conditions used in this experiment (Figure 2A). Nevertheless, these data showed that the enzyme retained activity and indicated that NiFe-cofactor insertion had occurred and that the C-terminal extension was removed. Addition of 1 mM Ni^2+^ ions to the growth medium failed to increase enzyme activity of pro-HybC^ST^ or pro-HybC^§ST^ (data not shown).

### The AV → ST Exchange in Pro-HybC^ST^ and Pro-HybC^§ST^ Did Not Affect Dependence on Either the HybG Chaperone or the HybD Protease

HybG is the chaperone protein specific for delivery of the Fe(CN)_2_CO group into pro-HybC during maturation of Hyd-2 (Blokesch et al., 2001; Sawers and Pinske, 2017). Pro-HyaB on the other hand can receive the Fe(CN)_2_CO group from either HybG or HypC (Blokesch et al., 2001). To determine whether the specificity of pro-HybC^ST^ for HybG was in any way altered by the exchange of the AV to ST residues, we introduced all four plasmids into the *hybG* mutant CB18 (Figure 2B). No clearly defined activity band corresponding to Hyd-2 could be observed in extracts of the *hybG* mutant (Figure 2B). When three of these constructs were introduced into strain CB19, which lacked HypC, the Hyd-2 activity profile of each was similar to that when the same plasmids were introduced into FTD150 (compare Figures 2A,C). This result indicates that all variants retained their dependence on HybG for maturation.

The final step in hydrogenase large subunit maturation after nickel-insertion involves the proteolytic cleavage of the C-terminal peptide. This cleavage event has been proposed to result in a conformational change in the protein resulting in closure of the active site (Drapal and Böck, 1998; Böck et al., 2006; Senger et al., 2017). Each precursor polypeptide has a specific protease that recognizes, among other features, the presence of the nickel ion as a motif (Theodoratou et al., 2000a,b). Although exceptions exist (Albareda et al., 2017), generally, each [NiFe]-hydrogenase large subunit has a protease specific for that particular large-subunit precursor (Böck et al., 2006; Sawers, 2012). This fact implies recognition of not only the nickel ion but also particular amino acid motifs of the hydrogenase precursor polypeptide by the protease (see Kwon et al., 2018). In *E. coli*, HyaD is the maturation endoprotease that is specific for pro-HyaB, while HybD is specific for pro-HybC (Theodoratou et al., 2005). Consequently, testing endoprotease specificity provides a very clear means of asking the question whether, apart from the nickel ion, the C-terminal peptide is a key determinant for protease recognition. To answer this question, we introduced the plasmids encoding the four different pro-HybC derivatives into isogenic FTD150 strains lacking either the *hybD* (Figure 2D) or the *hyaD* (Figure 2E) genes and analyzed the Hyd-2 activity profiles of cell-free extracts derived from the strains after anaerobic growth in minimal-glucose medium. The results showed very clearly that neither exchange of the 15-amino acid C-terminal peptide of pro-HybC for that of pro-HyaB (pro-HybC^§^) nor introduction of the AV → ST exchanges (pro-HybC^ST^) affected the protease specificity of the precursor; all four derivatives retained an absolute dependence on HybD (Figure 2D) and all four retained activity in the *hyaD* mutant background, although, however, the pro-HybC^§ST^ derivative had reduced activity (Figure 2E). Together, these data indicate that the specificity neither for the chaperone HybG nor for the protease HybD was affected by the amino acid substitutions in the C-terminal peptide of pro-HybC. Furthermore, they suggest that neither protein binds to the C-terminus of pro-HybC behind Cys-549 (Cys-4 in Figures 1A,C).

### The Pro-HybC^§ST^ Variant Including a Complete Exchange of the 18-Amino Acid C-Terminus of HybC Interacts With HybD

**Figure 3:**
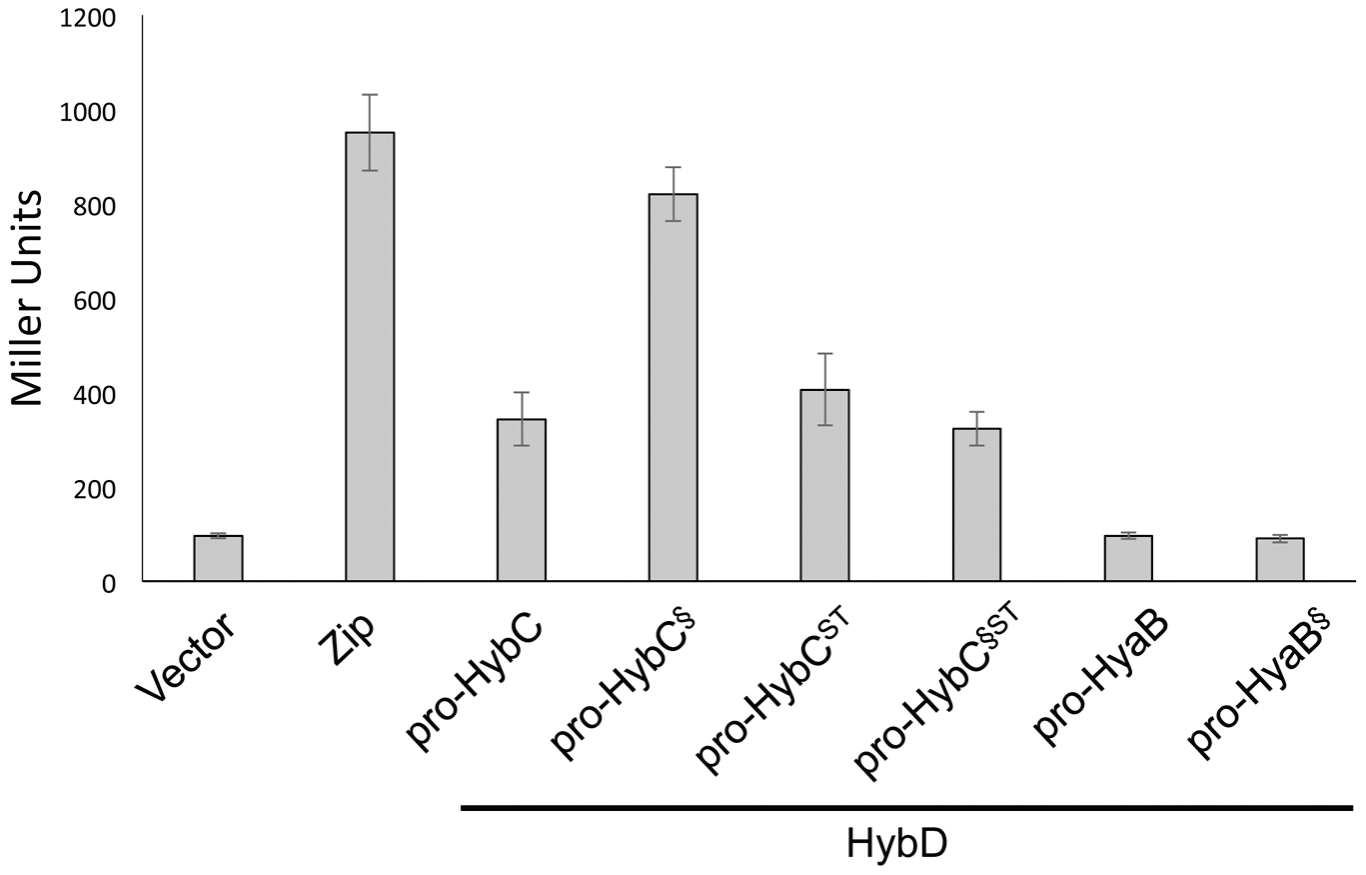
All four HybC variants interact with the endoprotease HybD. The histograms show the β-galactosidase enzyme activities of strain BTH101 carrying plasmids encoding T25 fusions of the indicated hydrogenase variants as well as T18 fused with HybD (see Section “Materials and Methods” for details). “Vector” shows the activity of the strain carrying empty vectors pUT18 and pKT25, while “Zip” shows the activity of the strain carrying the positive controls pT25-zip and pT18-zip (Karimova et al., 1998). Each histogram shows the mean plus standard deviation of the biological replicates, each performed in triplicate.

The results clearly show that pro-HybC^§ST^ could be matured into an active hydrogenase large subunit (HybC_proc_). Moreover, the results confirmed that this pro-HybC^§ST^ variant generated a lower Hyd-2 enzyme activity compared with pro-HybC^ST^. In order to demonstrate the protease specificity for HybD of all four pro-HybC derivatives, we performed bacterial two-hybrid analysis, whereby peptide T25 of adenylate cyclase was fused to the N-terminus of the HybC derivatives, while the protease HybD was fused with peptide T18 of adenylate cyclase (Karimova et al., 1998). The β-galactosidase enzyme activities measured for the empty vectors (pKT25 and pUT18) in the reporter strain BTH101 (Table 1) without insert, or pT25-zip together with pT18-zip, provided the negative or positive control values for the experiment, respectively (Figure 3). A clear interaction could be shown for HybD and pro-HybC, as reflected by the β-galactosidase enzyme activity of approximately 340 units. Similar activities were observed for both pro-HybC^ST^ and pro-HybC^§ST^, indicating both precursor proteins interacted with a similar affinity with HybD. Notably, pro-HybC^§^ had a significantly higher β-galactosidase activity (~ 820 units; Figure 3) in its interaction with HybD, suggesting an improved interaction between HybD and this precursor variant. A possible explanation for this finding is that the improved interaction may signify that HybD processes this variant more poorly.

In contrast to what was observed for fusions with pro-HybC, a fusion of the Hyd-1 large subunit precursor, pro-HyaB, or of pro-HyaB^§^, the Hyd-1 precursor with its C-terminus exchanged with that from pro-HybC, with the T25 adenylate cyclase fragment did not reveal any interaction with HybD (~95 units, similar to the negative control). This result indicates that the interaction between HybD and the HybC precursors was highly specific.

### Limited Proteolysis Studies Reveal That HybC_proc_ Has a More Compact Conformation Than Pro-HybC

**Figure 4:**
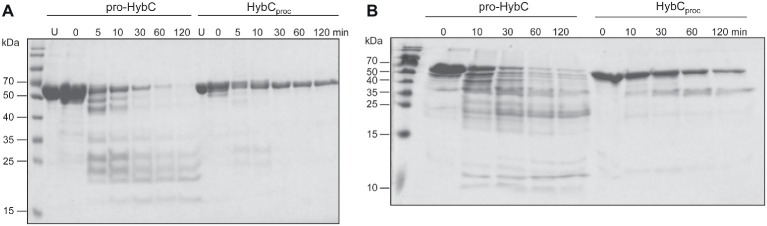
Different conformations of immature pro-HybC and mature HybC_proc_ revealed by limited proteolysis. Aliquots (100 μg protein) of purified His-tagged pro-HybC or HybC_proc_ were incubated at 37°C with 1 μg of trypsin **(A)** or chymotrypsin **(B)** and samples were removed at the indicated time points and separated in 10% (w/v) SDS-PAGE for **(A)**, or 12.5% (w/v) SDS-PAGE for **(B)**. After electrophoresis, the gels were stained with Coomassie Brilliant Blue. U, untreated, purified polypeptide. Molecular mass standards are shown in kDa. Limited proteolysis experiments were performed three times for each protease.

The findings of a recent CD-spectroscopic analysis have suggested that pro-HybC and HybC_proc_, the processed form of HybC lacking the 15-amino-acid long C-terminal peptide, have different conformations (Senger et al., 2017). To confirm and extend that observation, we undertook limited proteolysis experiments to gain insight into whether the genetic removal (HybC_proc_ in Figure 1B; Thomas et al., 2015) of the C-terminal peptide of pro-HybC indeed resulted in a more compact, “closed” conformation of the protein. The pro-HybC protein was isolated from a genetic background that lacked the *hybD* gene to ensure that no *in vivo* processing could occur. Studies were performed using both trypsin (Figure 4A) and chymotrypsin (Figure 4B), as well as using GluC (data not shown), and all three experiments showed an enhanced resistance of the purified HybC_proc_ species toward proteolysis compared with the unprocessed pro-HybC protein. After a 2 h incubation with trypsin, pro-HybC was almost completely cleaved (Figure 4A), while approximately 50% of HybC_proc_ remained uncleaved during the same period of incubation (Figure 4A). Similar observations were made for chymotrypsin treatment of the purified proteins (Figure 4B). Moreover, there were clearly fewer peptides visible in the 25–35 kDa mass range for the HybC_proc_ protein compared with pro-HybC (Figure 4A). Together, these findings indicated that HybC lacking the C-terminal peptide was more resistant toward proteolysis and therefore has a different, more “compact” conformation, compared with pro-HybC.

### HybG Protects Pro-HybC Against Proteolysis by Trypsin

**Figure 5:**
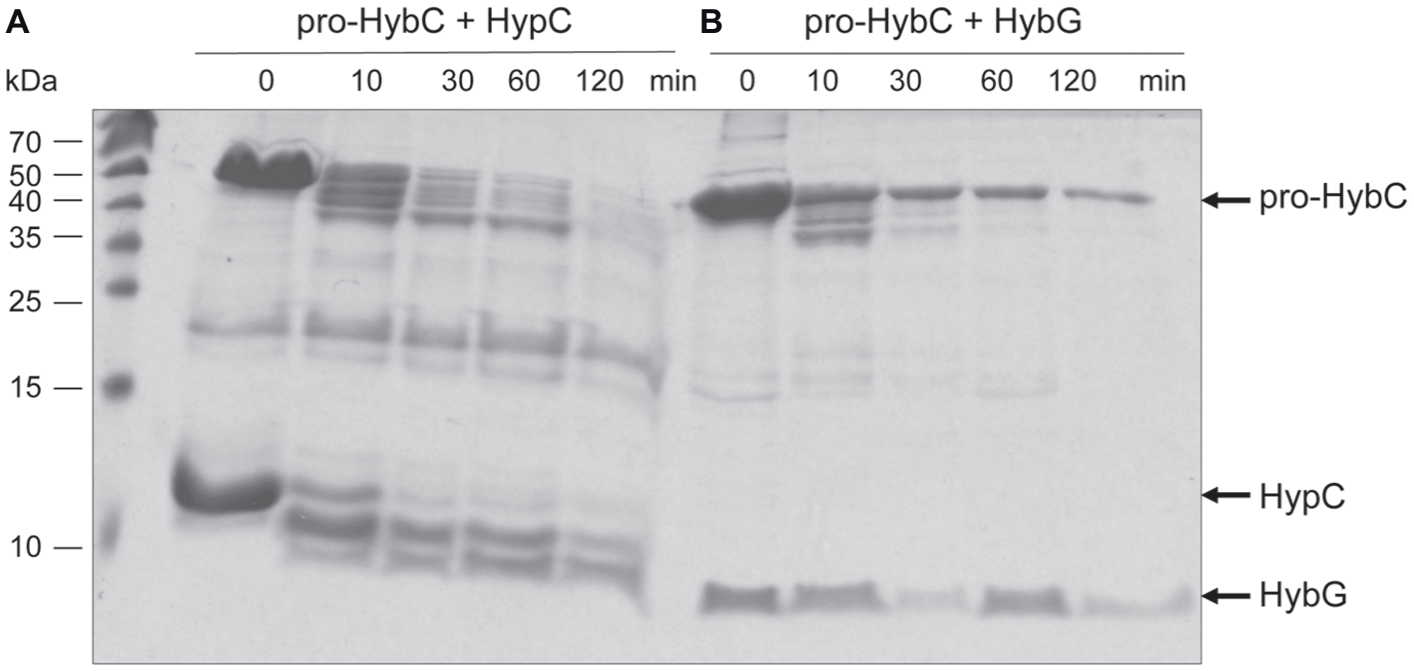
HybG protects pro-HybC against proteolytic attack by trypsin. The experiment shown in part **(A)** of Figure 4 was repeated under the same conditions but pro-HybC was incubated with 50 μg of either HypC **(A)** or HybG **(B)** prior to addition of trypsin. The migration positions of pro-HybC, HypC, and HybG are indicated on the right of the figure. Limited proteolysis experiments were performed three times.

HybG is required for the delivery of the Fe(CN)_2_CO group to pro-HybC (Blokesch et al., 2001) and HybG interacts with pro-HybC (Thomas et al., 2015; Senger et al., 2017). Our findings presented above also demonstrate that the pro-HybC variants with a C-terminal extension exchanged for that of pro-HyaB retained their dependence on HybG for maturation. These results strongly suggest that HybG, like the protease HybD, binds to and recognizes motifs in pro-HybC that are distinct from the C-terminal peptide, as has also been suggested based on the results of mutagenesis (Magalon and Böck, 2000) and recent structural studies (Kwon et al., 2018). To provide further support for this suggestion, we examined whether HybG, through its interaction with pro-HybC could offer protection against attack by trypsin (Figure 5). As a control, we performed at the same time an experiment in which HypC was incubated with pro-HybC. HypC does not specifically interact with pro-HybC (Blokesch et al., 2001; Thomas et al., 2015) and indeed the gel shown in Figure 5A reveals that HypC failed to protect pro-HybC against proteolysis by trypsin, with the degradation pattern being similar to that shown for pro-HybC alone (see Figure 4A). Full-length HypC was also degraded during the 2 h incubation with trypsin. In contrast, HybG clearly reduced the rate of degradation of pro-HybC (Figure 5B), suggesting that it either sterically hindered access of trypsin to the vulnerable sites, or altered the conformation of pro-HybC such that it was no longer accessible to the protease. Unfortunately, attempts to isolate pro-HybC^ST^ or pro-HybC^§ST^ for similar interaction analyses failed due to inherent instability of both variants (data not shown).

## Concluding Remarks

Our results support the conclusion that the C-terminal oligopeptide extension on the large subunit of Hyd-2 has a chaperone-like function (Theodoratou et al., 2005) and do not support the conclusion that it acts as a recognition motif for either the HybG or HybD hydrogenase maturation proteins. The two variable amino acids between Cys-4 and His-552 in HybC and HyaB of *E. coli*, and which are part of the conserved D-P-C-X-A-C-X-X-H motif that forms the C-terminus of most nickel hydrogenase large subunits, are interchangeable and not essential for either HybG or endoprotease specificity; however, the exchange from AV to ST in pro-HybC did impair the efficiency of maturation. It is likely that this effect is due to the proximity of these residues to the crucial fourth cofactor-coordinating cysteinyl residue (Cys-4), which ultimately bridges the two metals of the NiFe-cofactor in the active site, thus conferring activity upon the cofactor (Magalon and Böck, 2000; Beaton et al., 2018). The importance of the fourth cysteinyl residue in maturation was emphasized recently when the crystal structure of the immature precursor HyhL from *T. kodakarensis* revealed that a β-strand formed within the C-terminal extension and interacted with two β-strands in the N-terminus of the polypeptide to form a β-sheet structure. This conformational change displaced the Cys-4 residue away from the other three coordinating Cys residues (Kwon et al., 2018), consequently opening the active site to allow the Hyp maturation proteins to interact with and deliver the Fe(CN)_2_CO and nickel moieties to the precursor.

After endoproteolytic cleavage, the C-terminus is suggested to “flip”back forming a short α-helix closing the active site, thus allowing Cys-4 to bridge the two metals. This hypothesis is supported by the recently resolved structure of the mature HybC protein (Beaton et al., 2018; see also Figure 1C). Our limited proteolysis studies demonstrated that a major conformational change in the protein indeed occurs because HybC_proc_ became significantly more resistant to trypsin treatment when compared with pro-HybC. Moreover, interaction with HybG also protected the “open” conformation of the protein against trypsin, which corroborates our proposal that HybG possibly interacts with the N-terminal region of the protein, as has been shown for the nickel delivery protein HypA during its interaction with HyhL (Kwon et al., 2018). Moreover, these findings are in accord with HybG being unable to interact with HybC_proc_ (Thomas et al., 2015). Contrary to the proposed interaction of the HybD maturation endoprotease with the C-terminal extension (discussed in Kwon et al., 2018), the results presented in this communication indicate that the C-terminus is not a recognition motif for the hydrogenase-specific endoprotease, hence locating the recognition site(s) at a different place from the processing site. The two proteases HyaD and HybD do not recognize substrates other than their own large subunit precursors HyaB and HybC, respectively, and despite substitution of the complete C-terminal oligopeptide and the P2 and P3 sites from HyaB, pro-HybC^§ST^ retained its specificity for HybD.

## Data Availability Statement

The datasets generated for this study are available on request to the corresponding author.

## Author Contributions

CP, CT, and KN designed and performed the experiments and analyzed the data. CP and RS conceived the study, interpreted the data, and drafted the manuscript. All authors read and approved the manuscript.

### Conflict of Interest

The authors declare that the research was conducted in the absence of any commercial or financial relationships that could be construed as a potential conflict of interest.

## References

[ref1] Albareda M. Buchanan G. Sargent F. (2017). Identification of a stable complex between a [NiFe]-hydrogenase catalytic subunit and its maturation protease. FEBS Lett. 591, 338–347. 10.1002/1873-3468.12540, PMID: 28029689 PMC5299533

[ref2] Baba T. Ara T. Hasegawa M. Takai Y. Okumura Y. Baba M.. (2006). Construction of *Escherichia coli* K-12 in-frame, single-gene knockout mutants: the Keio collection. Mol. Syst. Biol. 2:2006.0008. 10.1038/msb4100050, PMID: 16738554 PMC1681482

[ref3] Ballantine S. P. Boxer D. H. (1985). Nickel-containing hydrogenase isoenzymes from anaerobically grown *Escherichia coli* K-12. J. Bacteriol. 163, 454–459. PMID: 3894325 10.1128/jb.163.2.454-459.1985 PMC219143

[ref4] Beaton S. E. Evans R. M. Finney A. J. Lamont C. M. Armstrong F. A. Sargent F.. (2018). The structure of hydrogenase-2 from *Escherichia coli*: implications for H_2_-driven proton pumping. Biochem. J. 475, 1353–1370. 10.1042/BCJ20180053, PMID: 29555844 PMC5902676

[ref5] Begg Y. Whyte J. Haddock B. (1977). The identification of mutants of *Escherichia coli* deficient in formate dehydrogenase and nitrate reductase activities using dye indicator plates. FEMS Microbiol. Lett. 2, 47–50. 10.1111/j.1574-6968.1977.tb00905.x

[ref6] Blokesch M. Magalon A. Böck A. (2001). Interplay between the specific chaperone-like proteins HybG and HypC in maturation of hydrogenases 1, 2, and 3 from *Escherichia coli*. J. Bacteriol. 183, 2817–2822. 10.1128/JB.183.9.2817-2822.2001, PMID: 11292801 PMC99498

[ref7] Böck A. King P. Blokesch M. Posewitz M. (2006). Maturation of hydrogenases. Adv. Microb. Physiol. 51, 1–71. 10.1016/S0065-2911(06)51001-X 17091562

[ref8] Casadaban M. J. (1976). Transposition and fusion of the *lac* genes to selected promoters in *Escherichia coli* using bacteriophage lambda and mu. J. Mol. Biol. 104, 541–555. 10.1016/0022-2836(76)90119-4, PMID: 781293

[ref9] Drapal N. Böck A. (1998). Interaction of the hydrogenase accessory protein HypC with HycE, the large subunit of *Escherichia coli* hydrogenase 3 during enzyme maturation. Biochemistry 37, 2941–2948. 10.1021/bi9720078, PMID: 9485446

[ref10] Fritsche E. Paschos A. Beisel H. G. Böck A. Huber R. (1999). Crystal structure of the hydrogenase maturating endopeptidase HybD from *Escherichia coli*. J. Mol. Biol. 288, 989–998. 10.1006/jmbi.1999.2719, PMID: 10331925

[ref11] Gollin D. J. Mortenson L. E. Robson R. L. (1992). Carboxyl-terminal processing may be essential for production of active NiFe hydrogenase in *Azotobacter vinelandii*. FEBS Lett. 309, 371–375. 10.1016/0014-5793(92)80809-U, PMID: 1516712

[ref12] Hedstrom L. (2002). Serine protease mechanism and specificity. Chem. Rev. 102, 4501–4524. 10.1021/cr000033x, PMID: 12475199

[ref13] Hormann K. Andreesen J. R. (1989). Reductive cleavage of sarcosine and betaine by *Eubaterium acidaminophilum via* enzyme systems different from glycine reductase. Arch. Microbiol. 153, 50–59. 10.1007/BF00277541

[ref14] Karimova G. Pidoux J. Ullmann A. Ladant D. (1998). A bacterial two-hybrid system based on a reconstituted signal transduction pathway. Proc. Natl. Acad. Sci. USA 95, 5752–5756. 9576956 10.1073/pnas.95.10.5752 PMC20451

[ref15] Kitagawa M. Ara T. Arifuzzaman M. Ioka-Nakamichi T. Inamoto E. Toyonaga H.. (2005). Complete set of ORF clones of *Escherichia coli* ASKA library (a complete set of *E. coli* K-12 ORF archive): unique resources for biological research. DNA Res. 12, 291–299. 10.1093/dnares/dsi012, PMID: 16769691

[ref16] Kwon S. Watanabe S. Nishitani Y. Kawashima T. Kanai T. Atomi H.. (2018). Crystal structures of a [NiFe] hydrogenase large subunit HyhL in an immature state in complex with a Ni chaperone HypA. Proc. Natl. Acad. Sci. USA 115, 7045–7050. 10.1073/pnas.1801955115, PMID: 29915046 PMC6142260

[ref17] Lacasse M. J. Zamble D. B. (2016). [NiFe]-hydrogenase maturation. Biochemistry 55, 1689–1701. 10.1021/acs.biochem.5b01328, PMID: 26919691

[ref18] Laemmli U. (1970). Cleavage of structural proteins during the assembly of the head of bacteriophage T4. Nature 227, 680–685. 10.1038/227680a0, PMID: 5432063

[ref19] Lowry O. Rosebrough N. Farr A. Randall R. (1951). Protein measurement with the Folin phenol reagent. J. Biol. Chem. 193, 265–275. PMID: 14907713

[ref20] Magalon A. Böck A. (2000). Analysis of the HypC-HycE complex, a key intermediate in the assembly of the metal center of the *Escherichia coli* hydrogenase 3. J. Biol. Chem. 275, 21114–21120. 10.1074/jbc.M000987200, PMID: 10783387

[ref21] Menon N. K. Robbins J. Vartanian M. Patil D. Peck H. D. Jr. Menon A. L.. (1993). Carboxy-terminal processing of the large subunit of [NiFe] hydrogenases. FEBS Lett. 331, 91–95. 10.1016/0014-5793(93)80303-C, PMID: 8405419

[ref22] Miller J. H. (1972). Experiments in molecular genetics. Cold Spring Harbor, NY: Cold Spring Harbor Laboratory.

[ref23] Pinske C. (2018). The ferredoxin-like proteins HydN and YsaA enhance redox dye-linked activity of the formate dehydrogenase H component of the formate hydrogenlyase complex. Front. Microbiol. 9:1238. 10.3389/fmicb.2018.01238, PMID: 29942290 PMC6004506

[ref24] Pinske C. Jaroschinsky M. Linek S. Kelly C. L. Sargent F. Sawers R. G. (2015). Physiology and bioenergetics of [NiFe]-hydrogenase 2-catalyzed H_2_-consuming and H_2_-producing reactions in *Escherichia coli*. J. Bacteriol. 197, 296–306. 10.1128/JB.02335-14 25368299 PMC4272588

[ref25] Pinske C. Jaroschinsky M. Sargent F. Sawers G. (2012). Zymographic differentiation of [NiFe]-hydrogenases 1, 2 and 3 of *Escherichia coli* K-12. BMC Microbiol. 12:134. 10.1186/1471-2180-12-134, PMID: 22769583 PMC3431244

[ref26] Pinske C. Krüger S. Soboh B. Ihling C. Kuhns M. Braussemann M.. (2011). Efficient electron transfer from hydrogen to benzyl viologen by the [NiFe]-hydrogenases of *Escherichia coli* is dependent on the coexpression of the iron-sulfur cluster-containing small subunit. Arch. Microbiol. 193, 893–903. 10.1007/s00203-011-0726-5, PMID: 21717143

[ref27] Redwood M. D. Mikheenko I. Sargent F. Macaskie L. (2008). Dissecting the roles of *Escherichia coli* hydrogenases in biohydrogen production. FEMS Microbiol. Lett. 278, 48–55. 10.1111/j.1574-6968.2007.00966.x, PMID: 17995952

[ref28] Rossmann R. Maier T. Lottspeich F. Böck A. (1995). Characterisation of a protease from *Escherichia coli* involved in hydrogenase maturation. Eur. J. Biochem. 227, 545–550. 10.1111/j.1432-1033.1995.tb20422.x, PMID: 7851435

[ref29] Rossmann R. Sauter M. Lottspeich F. Böck A. (1994). Maturation of the large subunit (HYCE) of *Escherichia coli* hydrogenase 3 requires nickel incorporation followed by *C*-terminal processing at Arg537. Eur. J. Biochem. 220, 377–384. 10.1111/j.1432-1033.1994.tb18634.x, PMID: 8125094

[ref30] Sambrook J. Fritsch E. F. Maniatis T. (1989). Molecular cloning: A laboratory manual. Cold Spring Harbor, New York: Cold Spring Harbor Laboratory Press.

[ref31] Sawers R. G. (2012). “Hydrogenase maturation endopeptidase” in Handbook of proteolytic enzymes. 3rd Edn. Chapter 70. eds. Rawlings N. Salvesen G. (Cambridge, Massachusetts, USA: Academic Press), 294–296.

[ref33] Sawers R. G. Pinske C. (2017). “Insights into [NiFe]-hydrogenase active site metallocluster assembly” in Encyclopedia of inorganic and bioinorganic chemistry – Metalloprotein site assembly, Chapter eibc2484. eds. Johnson M. K. Scott R. A. (Hoboken, New Jersey, USA: J. Wiley and Sons).

[ref34] Schechter I. Berger A. (1967). On the size of the active site in proteases. I. Papain. Biochem. Biophys. Res. Commun. 27, 157–162. 10.1016/S0006-291X(67)80055-X, PMID: 6035483

[ref35] Senger M. Stripp S. T. Soboh B. (2017). Proteolytic cleavage orchestrates cofactor insertion and protein assembly in [NiFe]-hydrogenase biosynthesis. J. Biol. Chem. 292, 11670–11681. 10.1074/jbc.M117.788125, PMID: 28539366 PMC5512064

[ref36] Soboh B. Pinske C. Kuhns M. Waclawek M. Ihling C. Trchounian K.. (2011). The respiratory molybdo-selenoprotein formate dehydrogenases of *Escherichia coli* have hydrogen: benzyl viologen oxidoreductase activity. BMC Microbiol. 11:173. 10.1186/1471-2180-11-173, PMID: 21806784 PMC3160892

[ref37] Soboh B. Stripp S. T. Bielak C. Lindenstrauß U. Braussemann M. Javaid M.. (2013). The [NiFe]-hydrogenase accessory chaperones HypC and HybG of *Escherichia coli* are iron- and carbon dioxide-binding proteins. FEBS Lett. 587, 2512–2516. 10.1016/j.febslet.2013.06.055, PMID: 23851071

[ref38] Theodoratou E. Huber R. Böck A. (2005). [NiFe]-hydrogenase maturation endopeptidase: structure and function. Biochem. Soc. Trans. 33, 108–111. 10.1042/BST0330108, PMID: 15667279

[ref39] Theodoratou E. Paschos A. Magalon A. Fritsche E. Huber R. Böck A. (2000a). Nickel serves as a substrate recognition motif for the endopeptidase involves in hydrogenase maturation. Eur. J. Biochem. 267, 1995–1999. 10.1046/j.1432-1327.2000.01202.x 10727938

[ref40] Theodoratou E. Paschos A. Mintz-Weber S. Böck A. (2000b). Analysis of the cleavage site specificity of the endopeptidase involved in the maturation of the large subunit of hydrogenase 3 from *Escherichia coli*. Arch. Microbiol. 173, 110–116. 10.1007/s002039900116 10795682

[ref41] Thomas C. Muhr E. Sawers R. G. (2015). Coordination of synthesis and assembly of a modular membrane-associated [NiFe]-hydrogenase is determined by cleavage of the *C*-terminal peptide. J. Bacteriol. 197, 2989–2998. 10.1128/JB.00437-15, PMID: 26170410 PMC4542169

